# European Veterinary Institutions1Read before the annual meeting of the American Veterinary Medical Association, New York, September, 1899.

**Published:** 1899-10

**Authors:** James B. Paige

**Affiliations:** Amherst, Mass.


### EUROPEAN VETERINARY INSTITUTIONS.[1]

By James B. Paige, D.V.S.,
AMHERST, MASS.

In travelling through Europe, studying its geographical position, its history, its resources, and the people; observing its agriculture, its animal industry, and immense armies; visiting its veterinary colleges and its veterinarians, one detects without difficulty

[1] Read before the annual meeting of the American Veterinary Medical Association, New York, September, 1899.

the incentive which has induced the different European governments to establish, equip, and maintain numerous veterinary institutions.

In the early centuries wars of conquest were frequent between the people of these countries, creating the necessity for large standing armies with artillery and cavalry. There were also frequent periodic invasions of devastating animal plagues, which brought poverty to the people and at times threatened ruin to the nations.

To provide educated men capable of directing the proper care of the horses in the army, to administer intelligent treatment in case of disease, and to investigate the nature and to arrest the spread of the animal plagues, these institutions were founded.

We find the same condition of affairs existing at the present time that existed more than a century ago, when the majority of the present colleges were founded.

There are now, as then, the large standing armies, with many horses to be cared for. Animal plagues, which devastated the flocks and herds in earlier times, still make their invasions. The proximity of the different countries, the density of the population, and the increased facilities for transportation with the consequent increase in traffic in livestock, make present conditions favorable for the rapid dissemination and propagation of contagious diseases, which are also much more difficult to control and arrest.

The same institutions which, more than a century and a half ago, sent out men to check these animal pests and to protect and develop the animal industry of the country, are still active in fitting men for the same work. One needs only to read the accounts of the invasions and results of the outbreaks of the animal plagues on the Continent to realize that in former times there existed a necessity for the establishment of schools in which men might receive instruction relative to these diseases.

A translation from an official historical paper by Secretary Grob, of Zürich, entitled, "Zur Geschicte der Thierarzneischule des Kantons Zürich," bears upon this point. In speaking of the history of the institution from 1820 to 1834, he says: "The education of the veterinarians at the beginning of the century was badly arranged for. It consisted simply of the rapid teaching of certain practical operations by a practising master or teacher. Frequently the father was the teacher of his sons. The correct diagnosis of disease was as little to be expected from these practitioners as was a comprehensive knowledge of the course of the same. It happened, therefore, that the country was continually visited by animal plagues.

At short intervals the whole of Europe was overrun with rinderpest, causing enormous losses. This condition created the necessity for skilled veterinarians, and the idea of founding a veterinary school gradually found expression."

Billings,[1] in writing of the school founded at Lyons, France, in 1762, says : "It soon acquired a Continental celebrity, and among the students of the first year we find the names of three Danes, three Swedes, three Austrians, three Prussians, three Sardinians, and ten Swiss, all sent to study the elements of the new medicine by and at the expense of their respective governments."

During the quarter century following the opening of the first veterinary college at Lyons, in 1762, we note the successive establishment of many veterinary institutions by the different Continental governments. The school at Alfort came three years after that at Lyons. The founding of the one at Toulouse was considered in 1769, but was not organized until 1825. The one at Vienna dates back to 1767; Copenhagen, 1773; Dresden, 1774; Hanover, 1778; Berlin, 1790, and the one at Munich the same year. Organized since that date we have the following: Stüttgart, 1821; Brussels, 1832; Utrecht, Holland, 1819; Bern, 1805; Zurich, 1820. Japan established one at Tokio in 1880. Portugal has one at Lisbon; Russia has no less than four of comparatively recent date; Spain has three or four, while Italy boasts of no less than nine, located in Boulogne, Milan, Modena, Parma, Pisa, Turin, Camerino, Perugia, and Urbino; that at Turin is the oldest, dating its foundation back to the year 1769.

Nearly, if not all, the institutions mentioned are in existence and actively engaged at the present time in the education of veterinarians for government positions, as teachers in veterinary colleges or agricultural schools, or as inspectors in abattoirs, in quarantine stations, or for the armies, or as private practitioners. The different governments recognize the advantages to be gained by having qualified men to look after these places. The demand for educated men for these positions is continually growing, and, considering the cost of living on the Continent, the income from them is good. Consequently, we find the different colleges well filled with students.

Having visited and inspected the institutions at Brussels, Alfort, Lyons, Bern, Zurich, Stüttgart, Dresden, Munich, Berlin, Vienna, and Budapest, I wish to call your attention to some of those things which proved most interesting to me.

[1] Relation of Animal Diseases to the Public Health.

The most striking features of these European colleges are their locations and grounds. Almost without exception they are located in the thickly settled portions of the largest cities. In some instances, as in Berlin, Dresden, and Budapest, quite central in the older portions of the cities. Notwithstanding this, they all have large grounds, surrounded by high walls or fences in true European style. The grounds at Berlin are said to be the largest occupied by any of the schools, containing not less than six acres. My impression is that those at Alfort are quite as extensive, if not larger. In this respect these two institutions are exceptions to the others mentioned, the average area of most of the college grounds being from three to four acres. These are always nicely laid out and beautified by trees and beds of flowers, pieces of statuary, fountains, etc. The locations and arrangements of the buildings, the walks and drives, are such as add beauty to the surroundings without sacrificing in respect to convenience. Almost without exception one finds on all the college grounds an artistic grouping of trees, shrubs, and flowering plants; but in no place are they to be found in such abundance as in the botanical gardens at Alfort and Lyons. Here we observe that considerable tracts of land have been set aside for these gardens, and in them are collections of several thousands of varieties of plants and shrubs. There are those varieties that yield some of the most valuable drugs as well as those prized for their foliage and bloom. These botanical gardens are made use of by the students in connection with their work in botany and materia medica.

On account of the situation of the grounds at Lyons a peculiar custom prevails there among the faculty and students in regard to the use of the garden. The city itself is divided into three parts by the rivers Rhone and Sôane, which flow from the north diagonally through it, converging and uniting at a point just outside the city limits. On the west side, starting back a short distance from the Saône, rising to the height of several hundred feet, is a steep, rocky hill. Between the base and the river, upon the level portion of land, stand the college buildings. In the rear of these, extending well up toward the crest of the hill, is the botanical garden. This has been formed into terraces, four or five in number, connected by winding paths and steps and provided with seats. These different levels are laid out with walks and beautified with beds of flowers. The students of the first class are allowed to use the first or lowest terrace for a promenade or rendezvous; those of the second year the one above, while those more advanced have access to those still higher, the upper level being reserved for the use of the faculty. Members of lower classes are not allowed to occupy terraces assigned for the use of students in higher classes.

The buildings of the Continental institutions are, almost without exception, constructed of brick, large and commodious, and very substantially built. Even at some of the oldest colleges one may see, in a good state of preservation, the original structures erected at the time the institutions were founded—buildings that look as if they were built to last until the end of time, and, judging from their present condition, one might be justified in inferring that the end was still a long way off.

The older buildings, particularly the stables, although large and substantially built, are, from our present knowledge of sanitary stable construction, very defective, usually only one story in height, with very small windows and doors, without any provision for proper drainage, ventilation, or lighting. They are, perhaps, most defective as hospitals, from the fact that their arrangement allows of little separation of patients. Horses with influenza, strangles, or pneumonia are stabled along with those under treatment for curb, spavin, or colic. Attempts to overcome this difficulty have been made in some instances by remodelling old buildings or by the erection of new ones. One frequently finds located in some remote corner of the grounds, or in all cases at a considerable distance from all other buildings, a small isolation box-stall, or small stable with several box-stalls separated by brick partitions. These are used for the segregation of patients with dangerous contagious diseases, such as horses with glanders or cattle with foot-and-mouth-disease, etc. In the centre of the grounds one finds, near the inside of the hedged square, an octagon-shaped isolation box-stall, which reminds one of a large well-house more than it does of a box horse-stall.

The practical application of the results of scientific investigations in bacteriology and pathology in the suppression and prevention of animal diseases during the last decade has given a mighty impetus to veterinary science in all Europe. The results accomplished have been appreciated by the governments as well as the individual. In consequence we find that the different governments have been liberal with the colleges, providing funds for the erection of new buildings, furnishing new equipment, establishing new departments, at the same time strengthening the old ones.

We see everywhere the effect of this liberality toward the colleges. At nearly every one we find one or more modern buildings, while at Bern and Budapest all the buildings are new. Alfort and Stüttgart have new and elegant quarters for chemistry, anatomy, zoölogy, and physiology; Lyons and Berlin, modern bacteriological and pathological laboratories, and, in addition, at Berlin, Prof. Fröhner's new surgery clinic, with stalls, forge, operating-hall, and laboratory for microscopical and chemical examinations, all constructed and arranged according to the most modern ideas relative to antisepsis and prophylaxis. Dresden has its elegant administration building and museums, and at Munich we find a new chemistry and physiological laboratory. The latest acquisition at Munich, just nearing completion, is the new clinic and administration building, for the construction of which the Bavarian government, three or four years ago, appropriated 500,000 marks ($125,000).

The most unique, if not the most satisfactory, addition in the line of buildings is to be seen at Brussels—a stable sufficiently large to accommodate twenty or twenty-five horses, constructed wholly of iron. The frame is constructed of trestle-work, in appearance not unlike a modern iron railroad bridge; this is covered with a thin, corrugated sheet-steel. The floors are of cement. This form of construction provides for easy and effective disinfection and protection from fire, but it has one very marked disadvantage that renders it practically worthless for winter use. When filled with animals the condensation of moisture, coming from the bodies of the animals, upon the cold steel walls and ceilings is so great that under certain atmospheric conditions it trickles down the walls or falls in drops from the ceilings upon the backs of the animals below.

(To be continued.)

